# 17β-Estradiol Is Involved in the Sexual Dimorphism of the Immune Response to Malaria

**DOI:** 10.3389/fendo.2021.643851

**Published:** 2021-03-26

**Authors:** Luis Antonio Cervantes-Candelas, Jesús Aguilar-Castro, Fidel Orlando Buendía-González, Omar Fernández-Rivera, Teresita de Jesús Nolasco-Pérez, Monserrat Sofía López-Padilla, David Roberto Chavira-Ramírez, Martha Legorreta-Herrera

**Affiliations:** ^1^ Laboratorio de Inmunología Molecular, Unidad de Investigación Química Computacional, Síntesis y Farmacología en Moléculas de Interés Biológico, División de Estudios de Posgrado e Investigación, Facultad de Estudios Superiores Zaragoza, Universidad Nacional Autónoma de México, Ciudad de México, Mexico; ^2^ Departamento de Biología de la Reproducción, Instituto Nacional de Ciencias Médicas y Nutrición Salvador Zubirán (INCMNSZ), Ciudad de México, Mexico

**Keywords:** malaria, 17β-estradiol, immune response, IFN-γ, TNF-α, IL-10, *P. berghei* ANKA, sexual dimorphism

## Abstract

Malaria is the leading cause of parasitic infection-related death globally. Additionally, malaria-associated mortality is higher in men than in women, and this sexual dimorphism reflects differences in innate and adaptive immune responses that are influenced by sex hormones. Normally, females develop more robust immune responses against parasites than males. However, most clinical and laboratory studies related to the immune response to malaria do not consider sex as a variable, and relatively few studies have compared the sex-dependent role of 17β-estradiol in this process. In this study, we decreased *in vivo* the levels of 17β-estradiol by gonadectomy or administered 17β-estradiol to intact or gonadectomized male and female CBA/Ca mice infected with *Plasmodium berghei* ANKA. Subsequently, we assessed the effects of 17β-estradiol on parasite load; the percentages of different immune cells in the spleen; the plasma levels of antibodies and pro- and anti-inflammatory cytokines; and the mRNA expression levels of cytokine-encoding genes in the brain. The results showed that the administration of 17β-estradiol increased parasitemia and decreased body weight in intact female mice. Moreover, intact females exhibited higher levels of CD8^+^ T cells and lower levels of NK1.1^+^ cells than their male counterparts under the same condition. Gonadectomy increased IFN-γ and decreased TNF-α concentrations only in intact female mice. Additionally, IL-10 levels were higher in intact females than in their male counterparts. Finally, the mRNA expression levels of cytokines coding genes in the brain showed a dimorphic pattern, i.e., gonadectomy upregulated *Tnf*, *Il1b*, and *Il10* expression in males but not in females. Our findings explain the sexual dimorphism in the immune response to malaria, at least in part, and suggest potential sex-dependent implications for the efficacy of vaccines or drugs targeting malaria.

## Introduction

Malaria is the leading cause of parasitic disease-related mortality worldwide (1). A marked sexual dimorphism is characteristic in malaria; males develop more severe symptoms and exhibit higher mortality rates than females (2, 3). However, sex is rarely recognized as a variable in both human clinical studies and experimental malaria models; therefore, differences between male and female responses are rarely reported. Since sex hormones are responsible for the most critical differences between sexes, the higher rate of male mortality suggests female hormones exert a protective effect against *Plasmodium*, the malaria parasite (4). Accordingly, 17β-estradiol receptors (ERα and ERβ) are expressed in the immune response cells such as T cells, B cells (CD19^+^), dendritic cells, macrophages, and natural killer (NK1.1^+^) cells (5). All these cells are involved in *Plasmodium* elimination suggesting that 17β-estradiol modulates immunocompetence.

17β-estradiol participates in the development and maturation of immune cells and diverse signaling pathways of the innate and adaptive immune responses (5). ERs are transcription factors that form complexes at gene-regulatory elements and promote epigenetic modifications and gene transcription (6). They can also activate steroid signaling in the membrane through the G protein-coupled ER, inducing rapid responses (7). Consequently, 17β-estradiol modulates different signaling pathways in B cells, T cells, dendritic cells, NK cells and macrophages (8–11).

The research about the participation of 17β-estradiol in malaria immune response has driven to controversial conclusions. Benten et al. documented that 17β-estradiol suppresses the development of immunity to *Plasmodium chabaudi* in C57BL/10 mice. However, the administration of 17β-estradiol to immune mice does not modify parasitemia (12). In contrast, Libonati et al. informed that 17β-estradiol decreases parasitemia but increases cerebral malaria development in CBA/Ca female mice infected with *Plasmodium berghei* ANKA (13). On the other hand, Klein et al. showed that 17β-estradiol increases IL-10 and INF-γ levels and promotes the production of IgG1 in C57Bl/6 female mice (14). The results of studies related to the effects of 17β-estradiol in the immune response to malaria-infected individuals have been inconsistent, and the sex-dependent effects of 17β-estradiol remain poorly understood. In this study, we analyzed the role of 17β-estradiol in the sexual dimorphism of the immune response against *Plasmodium*. For this, we either decreased the level of this steroid by gonadectomy or administered it to intact or castrated male or female CBA/Ca mice. Then, we infected all mice with the lethal *Plasmodium berghei* ANKA [this host-parasite combination constitutes the gold model for cerebral malaria and resembles the infection with the lethal *Plasmodium falciparum* in humans (15)]. We analyzed the effects on parasite load; the percentages of immune response cells in the spleen; the plasma levels of antibodies; and pro- and anti-inflammatory cytokines. Finally, because the main malaria complication that drives death is cerebral malaria, we analyzed the mRNA expression levels of cytokine-encoding genes in the brain.

## Materials and Methods

### Mice, Parasites, and Infection

CBA/Ca mice were a generous gift from Dr. William Jarra (National Institute for Medical Research, London, UK). The mice were raised, fed, and maintained in a controlled atmosphere (filtered air, autoclaved drinking water, food, bed and jails; regulated 12-hour cycles of light and dark; three physical barriers before access to experimental mice, and periodically tested against specific pathogens) at the animal housing facilities of FES Zaragoza, Universidad Nacional Autónoma de México. All animal procedures were approved by the Institutional Care and Animal Use Committee of the University. Mice were euthanized by cervical dislocation after anesthesia with 5% sevoflurane (Abbot, Mexico City, México).

*Plasmodium berghei* ANKA parasites were a generous gift from Dr. William Jarra. The parasites were cryopreserved under liquid nitrogen. For parasite activation, one vial was thawed and immediately injected into one four-week-old mouse. When parasitemia attained 20%, parasitized blood was extracted in PBS/heparin. Total erythrocyte count was assessed in a Neubauer chamber and the percentage of parasitized red blood cells was evaluated microscopically in Giemsa stained blood smears. To prepare the inoculum, blood was diluted with PBS to obtain 1×10^4^ *Plasmodium berghei* ANKA*-*parasitized erythrocytes/mL. Then, 100 µL of such inoculum were injected in the caudal vein to each mouse. We used this route to be sure all inoculated parasites were in blood at the same time.

### Orchiectomy and Ovariectomy

Orchidectomy was performed as we previously described (16). Four- week-old CBA/Ca male mice were anaesthetized by using a mixture of Ketamine: Xylazine ((80 mg/Kg: 8mg/Kg) (Phoenix Pharmaceutical Inc., St. Joseph, MO, USA). The scrotal fur was eliminated, and then scrotal incisions were made under aseptic conditions. Testes and epididymis were removed by electrocauterization, and incisions were sutured. For mice recovery, they were used after 4 weeks after surgery.

Ovariectomy. Four-week-old female mice were anaesthetized, and incisions were made in the lower abdomens under aseptic conditions. The ovaries were removed, and the abdominal muscle-wall incisions were sutured. Male and female mice were used 4 weeks after surgery.

### 17β-Estradiol Administration and Parasitemia

Male or female mice were subcutaneously injected with 545 µg of 17β-estradiol/kg of body weight (approximately 12 µg/mouse) in 50 µL of sesame oil (vehicle) twice a week for three weeks as previously described by Benten et al. (12). By using this 17β-estradiol concentration, we have previously showed 17β-estradiol effects on oxidative stress (17).

Parasitemia was evaluated in methanol-fixed, Giemsa-stained (Sigma-Aldrich, St. Louis, MO, USA) blood smears. The parasite load was quantified under a 100× oil immersion objective lens using a Carl Zeiss Standard 20 microscope (Carl Zeiss Ltd, Welwyn Garden City, UK). The number of parasitized red blood cells was evaluated in 50 fields. The parasitemia in each group was presented as the mean of parasitized erythrocytes ± SEM. The experiment was repeated twice per group (*n*= 10).

### Change in Body Mass

Mice were weighed daily from day 0 to day 8 post-infection using a semi-analytical weighing scale (Ohaus Parsippany, NJ, USA). The change in body mass was calculated as the percentage relative to day 0 (0%) in each group; the data are presented as means ± SEM.

### Body Temperature

Body temperature was measured daily at the same hour from day 0 to day 8 post-infection using an infrared thermometer (Thermofocus, 01500A/H1N1, Vedano Olana-Varese, Italy). The body temperature on day 0 was considered 0%; the body % temperature change was calculated as the percentage relative to day 0.

### Hemoglobin (Hb) Concentration

A small cut of the mice tail was made using a surgery cutter, then 2 µL of blood were collected using a micropipette. The excess of blood on the tip was carefully eliminated with tissue paper. Blood was immediately mixed with 498 µL of Drabkin’s reagent (Sigma-Aldrich). The optical density in this mixture was recorded at 540 nm by using a microplate reader (Multiskan GO, Termo Fisher Scientific, Inc, Waltham, MA, USA). The Hb concentration was calculated by using a commercial Hb standard (Sigma-Aldrich).

### Cytokine Quantification

Gonadectomized or intact male and female mice treated with 17β-estradiol were infected with *P. berghei* ANKA. On day 8 post-infection, mice were sacrificed by cervical dislocation. Blood from the heart was extracted and immediately transferred into heparinized tubes and centrifuged at 2000×g for 15 min. Plasma was separated and frozen at -70°C until use. The concentration of the cytokines IFN-γ, TNF-α, IL-2, IL-4, IL-5, IL-6, IL-10 and IL-17a were quantified using a cytometric bead array (BD Mouse Th1/Th2/Th17 cytokine CBA Kit Biosciences-Pharmingen, Heidelberg, Germany) according to the manufacturer’s protocol, except that the assay was performed in microtubes. The standard curve started at 0.625 pg/mL. The sensitivity attained was 0.9 ± 0.05 pg/mL, and the inter-assay variation was 5%.

### Antibody Quantification

*Plasmodium berghei* ANKA-specific antibodies were quantified using a previously described method (18). Briefly, plates were incubated overnight at 4°C with 10 µg/mL *P. berghei* antigen in carbonate buffer. The plates were then blocked using 3% skimmed milk in PBS for 2 h at 37°C, and incubated with 100 µL of 1:20 diluted plasma in PBS containing 0.02% milk for 1 h at 37°C, and then with a biotin-conjugated anti-mouse IgG or IgM monoclonal antibody (Zyme, San Francisco, CA, USA) diluted in PBS containing 0.02% skimmed milk and 0.05% Tween 20. Subsequently, the plates were incubated with streptavidin-horseradish peroxidase, and the stained was developed with 0.4 mg/mL O-phenylenediamine (Sigma-Aldrich) in citrate buffer (pH 5.0) with 0.03% hydrogen peroxide for 20 min. Absorbance was measured at 492 nm with a microplate reader (Multiskan GO, Termo Fisher Scientific, Inc.). Because no standard of known concentration was available for IgG or IgM, the results were expressed as OD 450 nm values and compared to an internal standard of normal CBA mouse plasma obtained from eight-week-old naive female or male mice. This internal standard provided a background value of nonspecific responsiveness to the lysate used.

### Quantification of Cell Subpopulations in the Spleen

The expression of cell surface markers was assessed by multicolor flow cytometry as previously described (16). Briefly, on day 8 post infection, mice were sacrificed, and their spleens were removed and pushed through a nylon mesh. Red blood cells were eliminated using lysis buffer (phosphate-buffered saline (0.15 M NH_4_Cl, IM KHCO_3_, 0.1 mM Na_2_EDTA, pH 7.2). The splenic cells were washed with PBS and stained with pre-calibrated dilutions of the following mouse-specific antibodies were used: FITC-conjugated anti-CD3, APC-conjugated CD4, PE-conjugated anti-CD8, APC-conjugated anti-CD19, biotin-conjugated anti-NK1.1, and PE-anti-Mac3 PE-conjugated streptavidin was used for biotin detection. All the antibodies were purchased from BioLegend (San Diego, CA, USA). Stained cells were quantified using a FACSAria II flow cytometer, and data were analyzed with FlowJo software.

### mRNA Expression of *Tnf*, *IL1b* and *Il10* in the Brain

The mRNA expression of cytokine-encoding genes was quantified by qPCR assay as previously described (16). Briefly, on day 8 post-infection, mRNA was extracted from the brain using TRIzol (Invitrogen, Carlsbad, CA, USA) followed by treatment with DNAse I (Invitrogen). To obtain cDNA, 1.5 µg of RNA was incubated for 1 h at 37°C with 0.5 µg of oligo (dT) primers (Promega, Madison, WI, USA), 200 U of MMLV-RT (Invitrogen), 0.5 mM dNTPs (Invitrogen), and 40 U of RNAse inhibitor. qPCR was performed on an ABI 7500 thermocycler (Applied Biosystems, Foster City, CA, USA). The following primers were used: *Tnf* forward 5’CGG CGT TCT TTG AGA TCC ATG C[FAM]G-3’, and reverse 5’CGT CGT AGC AAA CCA CCA AGT G3’; *Il10* forward 5’CGG TTC TGG ACA ACA TAC TGC TAAC [FAM] C3’, reverse 5’TGG ATC ATT TCC GAT AAG GCT TG3’; *Il-1b* forward 5’CAA CCA ACA AGT GAT ATT CTC CAT G3’, reverse 5’GAT CCA CAC TCT CCA GCT GCA3’; β-actin forward 5’CGG GTC AGG TAG TCT GTC AGG TCC [JOE] G3’, reverse 5’CTA TGC TCT CCC TCA CGC CAT C3’. Each reaction contained 1× PCR Master Mix (Invitrogen), 10 nM of each forward and reverse primers, and 1 µL of cDNA. Standard curves were prepared using serial cDNA dilutions. The assay was performed in triplicate. mRNA expression levels were normalized to that of β-actin and quantified using the 2^ΔΔCT^ method (ΔΔCT analysis, User Bulletin No.2, ABI Prism 7700 Applied Biosystems).

### Experimental Design

The gonad is the primary sex steroid-synthesizing tissue (19). To analyze the effect of 17β-estradiol on the sexual dimorphism in the immune response against *P. berghei* ANKA, the level of this steroid was downregulated *via* gonadectomy in male and female mice. Additionally, 17β-estradiol was also administered to groups of both intact or gonadectomized male or female mice.

4-week-old CBA/Ca mice (20 males and 20 females) were used. Mice were randomly divided into 4 groups for each sex (5 mice in each group). The first group was administered with vehicle; the second group was gonadectomized and treated with vehicle; the third group was treated with 17β-estradiol; and the fourth group was gonadectomized and treated with 17β-estradiol. Female mice were organized and treated in the same way. All mice were infected with *Plasmodium berghei* ANKA. The day of infection was considered day zero. Body weight change, temperature, and hemoglobin concentration were evaluated daily. Parasitemia was assessed from day 3 to 8 post-infection. On day 8 post-infection, all mice were sacrificed, and blood, spleen and brain were removed. Plasma was obtained from the blood and used to quantify cytokine and antibody levels; the spleen was disaggregated and processed to quantify cell populations. Finally, RNA was extracted from the brain analysis of mRNA expression *Tnf*, *Il1b* and *I110* genes. The whole study was performed in duplicate in two independent experiments using 5 mice per group (*n* = 10).

### Statistical Analysis

For data analysis, we evaluated whether data had a normal distribution by using the Shaphiro-Wilks test (*p* ≤ 0.05). Parasitemia, change in body mass, temperature change, and hemoglobin concentration exhibited a normal distribution. Therefore, we compared male versus female by using ANOVA with a 95% confidence interval. However, cell populations, cytokine concentration, mRNA expression, and antibody levels data did not exhibit a normal distribution. Therefore, we used Kruskal-Wallis with a post-hoc Dunn’s test; with a *p* ≤ 0.05. Statistical Analysis was performed in Statgrafics version XVI software (Statgraphics Technologies, Inc. The Plains, Virginia, USA). All the results are expressed as means ± SEM of two independent experiments using five mice in each (*n* = 10) mice per group.

## Results

### 17β-Estradiol Exerted a Sexually Dimorphic Effect on Parasitemia, Body Weight, Temperature, and Hemoglobin Concentrations in Mice Infected With *P. berghei* ANKA

Decreases in body weight, temperature, and hemoglobin concentrations are the main features of malaria-infected mice. To assess the role of 17β-estradiol in these variables, we measured body mass, body temperature, and hemoglobin concentrations of the mice daily. Because *P. berghei* ANKA is lethal in CBA/Ca mice and kills mice from day 9 to 11 after infection; we halted the experiment on day 8 post-infection.

**Figure 1:**
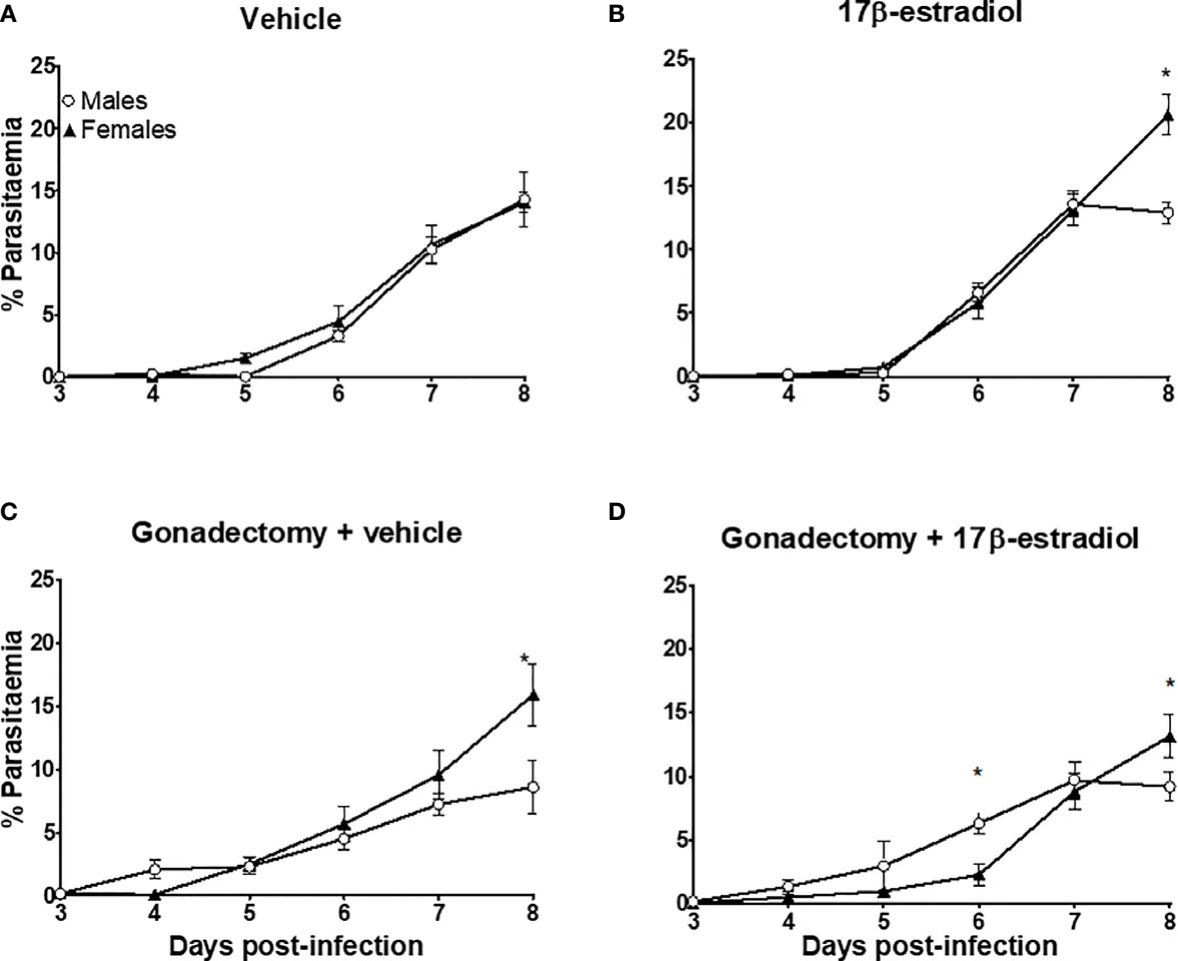
The dimorphic effect of 17β-estradiol on parasitaemia. Intact or gonadectomized male and female mice were treated with 17β-estradiol for three weeks and then infected with *P. berghei* ANKA. Parasitemia was evaluated daily from day 3 to 8 post-infection in thin blood smears stained with Giemsa. Intact male and female mice treated with vehicle **(A)**; intact male and female mice treated with 17β-estradiol **(B)**, Gonadectomized male and female mice treated with vehicle **(C)**, and Gonadectomized male and gonadectomized female mice treated with17β-estradiol **(D)**. Each point represents the mean ± SEM in each group. Data are representative of two independent experiments, each using five mice (*n* = 10). Asterisk indicate significant differences using ANOVA with a significance of <0.05.

Intact male and female mice exhibited similar parasitemia during the experiment **(** **Figure 1A** **)**. The administration of 17β-estradiol to intact mice significantly increased parasitemia in females on day 8 post-infection **(** **Figure 1B** **)**. Gonadectomy had a differential effect, it reduced parasitemia in male mice, while in females it increased, especially on day 8 post-infection **(** **Figure 1C** **)**. Interestingly, administration of 17β-estradiol to gonadectomized mice increased parasitemia in males but decreased it in females on day 6 post-infection **(** **Figure 1D** **)**.

**Figure 2:**
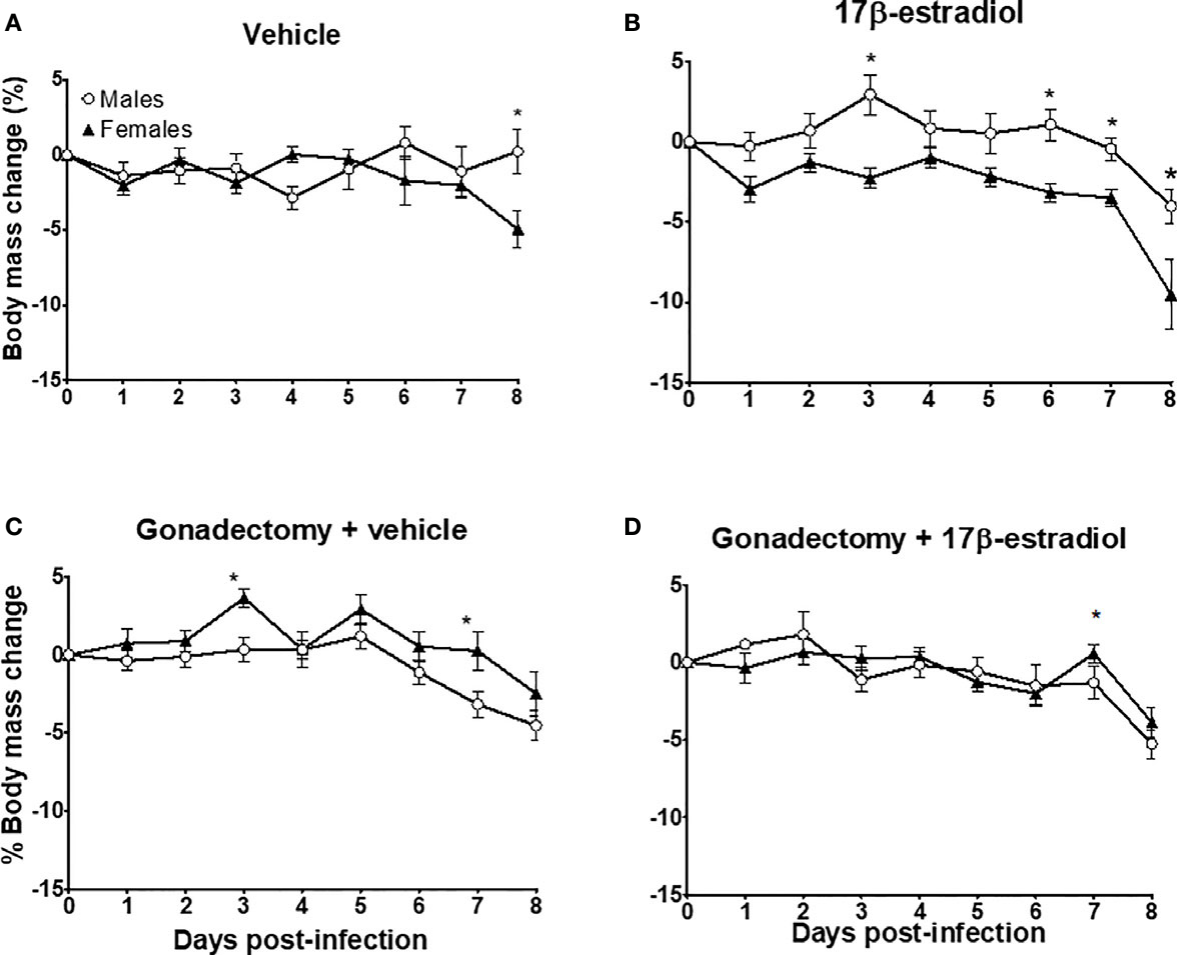
The dimorphic effect of 17β-estradiol on body mass change. Intact or gonadectomized male and female mice were treated with 17β-estradiol for three weeks and then infected with *P. berghei* ANKA. Mice were weighed daily. The weight on day 0 post-infection was recorded as 0% and the weight in each day was calculated related to day 0. Intact male and female mice treated with vehicle **(A)**; intact male and female mice treated with 17β-estradiol **(B)**, Gonadectomized male and female mice treated with vehicle **(C)**, and Gonadectomized male and female mice treated with17β-estradiol **(D)**. Each point represents the mean ± SEM. Each point represents the mean ± SEM in each group. Data are representative of two independent experiments, each using five mice (*n* = 10). Asterisk indicate significant differences using ANOVA with a significance of <0.05.

On day 0 (infection day), the mean body mass for males was 25.08 g ± 0.36, and for female mice was 21.5 g ± 0.18. Intact male and female mice exhibited similarities in body mass during the experiment, except by day 8 post-infection: females significantly decreased body mass while males increased it **(** **Figure 2A** **)**. The administration of 17β-estradiol to intact mice significantly increased the body mass in males; while in females, the opposite was observed, generating a dimorphic pattern during the experiment **(** **Figure 2B** **)**. Gonadectomy decreased the body mass in males but increased it in females also generating a dimorphic pattern **(** **Figure 2C** **)**. The administration of 17β-estradiol to gonadectomized mice reduced the differences between sexes and eliminated the dimorphic pattern **(** **Figure 2D** **)**. The above effects are all indicative of sexual dimorphism.

**Figure 3:**
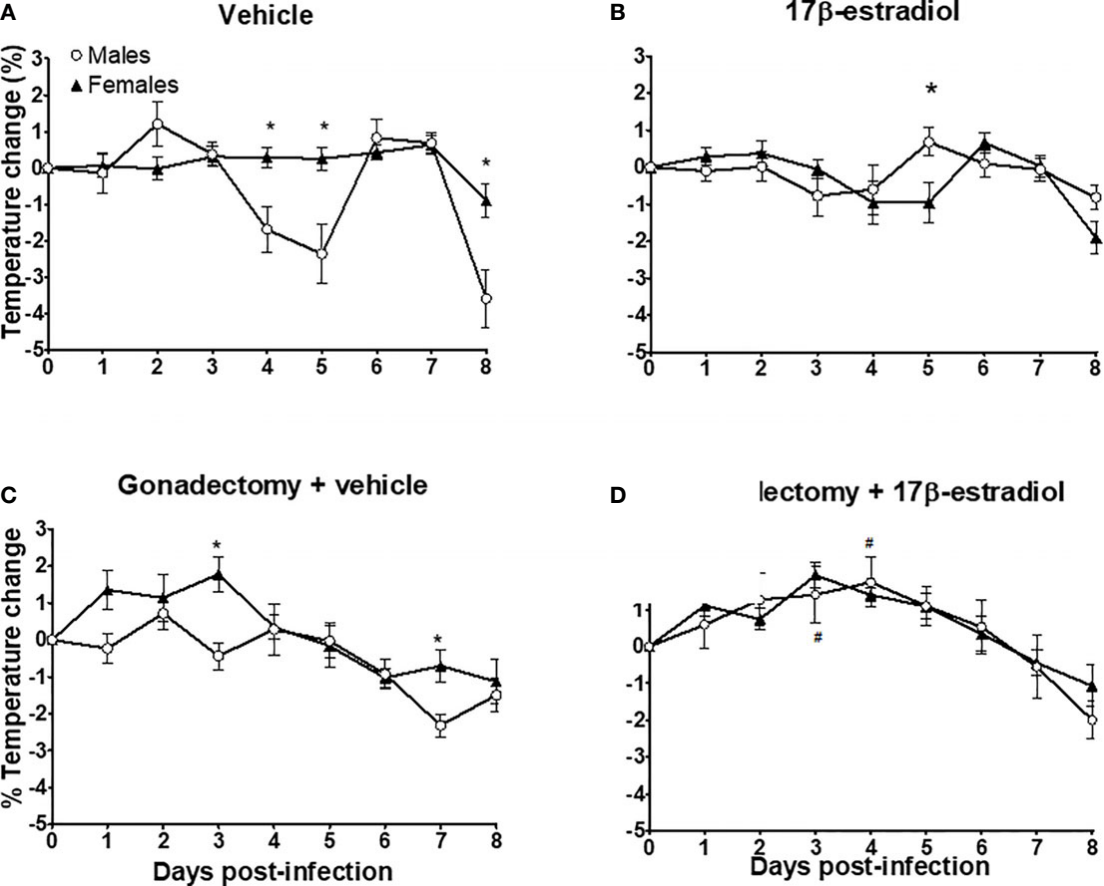
The dimorphic effect of 17β-estradiol on temperature change. Intact or gonadectomized male and female mice were treated with 17β-estradiol for three weeks and then infected with *P. berghei* ANKA. Body temperature was recorded daily. The temperature on day 0 post-infection was recorded as 0% and the temperature change in each day was calculated related to day 0. Intact male and female mice treated with vehicle **(A)**; intact male and female mice treated with 17β-estradiol **(B)**, Gonadectomized male and female mice treated with vehicle **(C)**, and Gonadectomized male and female mice treated with17β-estradiol **(D)**. Each point represents the mean ± SEM in each group. Data are representative of two independent experiments, each using five mice (*n* = 10). Asterisk indicate significant differences using ANOVA with a significance of <0.05. # denotes significant differences between gonadectomized male mice treated with vehicle and gonadectomized male mice treated with 17β-estradiol.

Intact females exhibited a better control in body temperature than intact males whose temperature showed a high variation **(** **Figure 3A** **).** The administration of 17β-estradiol to intact mice increased body temperature in males, eliminating the dimorphic pattern **(** **Figure 3B** **)**. Gonadectomy decreased temperature in male mice; while in female mice gonadectomy increased it, particularly during the first 3 days post-infection **(** **Figure 3C** **)**. Finally, the administration of 17β-estradiol to gonadectomized mice resulted in a significant increase in body temperature only in males, eliminating the dimorphic pattern (**Figure 3D**).

**Figure 4:**
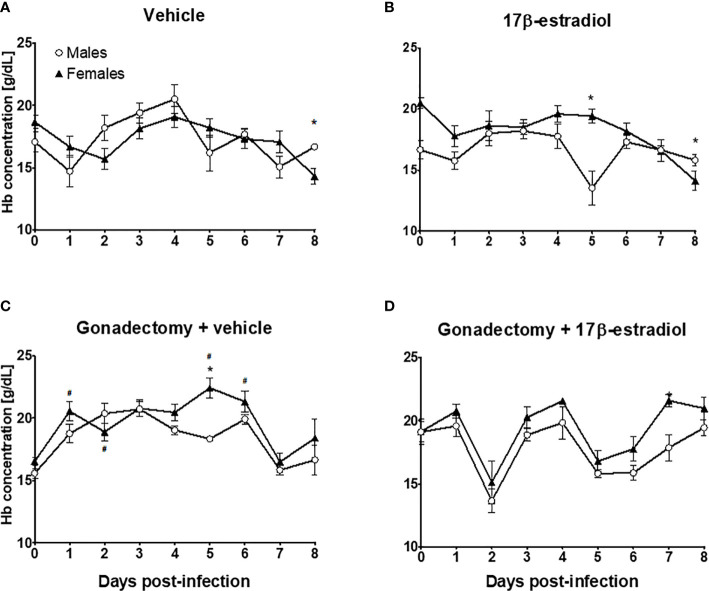
The dimorphic effect of 17β-estradiol on hemoglobin concentration. Intact or gonadectomized male and female mice were treated with 17β-estradiol for three weeks and then infected with *P. berghei* ANKA. Hemoglobin concentration was measured daily. Intact male and female mice treated with vehicle **(A)**; intact male and female mice treated with 17β-estradiol **(B)**, Gonadectomized male and female mice treated with vehicle **(C)**, and Gonadectomized male and female mice treated with17β-estradiol **(D)**. Each point represents the mean ± SEM in each group. Data are representative of two independent experiments, each using five mice (*n* = 10). Asterisk indicate significant differences using ANOVA with a significance of <0.05. # denotes significant differences between intact female mice treated with vehicle and gonadectomized female mice treated with vehicle.

Intact female and male mice exhibited similar Hb concentration during the experiment **(** **Figure 4A** **)**. The administration of 17β-estradiol to intact mice decreased the Hb concentration in males, particularly on day 5 post-infection **(** **Figure 4B** **)**. Gonadectomy increased Hb concentrations only in female mice **(** **Figure 4C** **)**. However, gonadectomized female mice treated with 17β-estradiol exhibited higher Hb levels than males in the same condition **(** **Figure 4D** **)**. These results indicated that the effects of 17β-estradiol treatment on Hb concentrations were sex-dependent.

### The Reduction in 17β-Estradiol Levels in Gonadectomized Animals Affected the Percentages of Immune Response-Related Cells in the Spleen of Mice Infected With *P. berghei* ANKA

**Figure 5:**
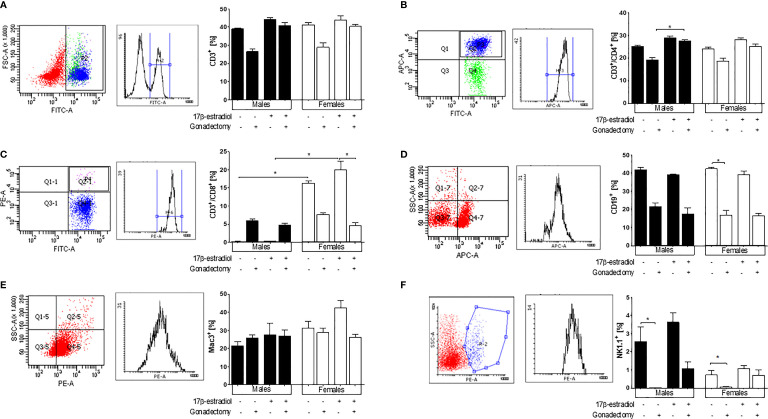
The effect of 17β-estradiol on immune subpopulations in the spleen of *Plasmodium berghei* ANKA-infected mice. Flow cytometry analysis of indicated immune cell populations in the spleen of intact or gonadectomized male or female mice treated with 17β-estradiol and infected with *P. berghei* ANKA. First panel representative dot plots of cytometric analysis, second panel represent the histogram and the graphic represent the percentage of the following cells: CD3^+^ **(A)**, CD3^+^/CD4^+^ **(B)**, CD3^+^/CD8^+^ **(C)**, B cells (CD19^+^) **(D)**, macrophages (Mac-3^+^) **(E)**, and natural killer (NK1.1^+^) cells **(F)**. Each bar represents the mean ± SEM in each group. Data are representative of two independent experiments, each using five mice (*n* = 10). Asterisks indicate significant differences between selected groups (*p≤* 0.05) calculated using Kruskal-Wallis test with a post -hoc Dunn’s test.

Because the spleen is the main organ where immune cells eliminate the *Plasmodium* parasite (20), we analyzed the effects of 17β-estradiol treatment on different subpopulations of immune cells in this organ. Gonadectomy decreased in trend both CD3^+^ and CD3^+^/CD4^+^ cells in male and female mice. However, 17β-estradiol treatment restored these populations only in males. Meanwhile, in intact mice, these subpopulations were not affected by 17β-estradiol treatment (**Figures 5A, B** **)**.

Both intact females and gonadectomized females exhibited significantly higher percentages of CD3^+^/CD8^+^ cells compared with intact males and gonadectomized males showing a dimorphic pattern. The administration of 17β-estradiol to intact male or female mice did not modify the levels of CD3^+^/CD8^+^ cells (**Figure 5C**).

Gonadectomy reduced the percentage of CD19^+^ cells in both sexes; however, 17β-estradiol administration did not restore this subpopulation in gonadectomized mice. No changes in the CD19^+^ cell percentages were observed in 17β-estradiol-treated intact mice (**Figure 5D**).

Neither 17β-estradiol administration nor gonadectomy affected the macrophage (Mac3^+^) population (**Figure 5E**). Finally, intact male mice exhibited higher percentages of population NK1.1^+^ cells than intact females, which represented a sexually dimorphic pattern. Gonadectomy reduced the percentage of this cell population in both sexes, while 17β-estradiol treatment reversed this effect only in females (**Figure 5F** **)**.

### 17β-Estradiol Differentially Modified Plasma Cytokine Concentrations

**Figure 6:**
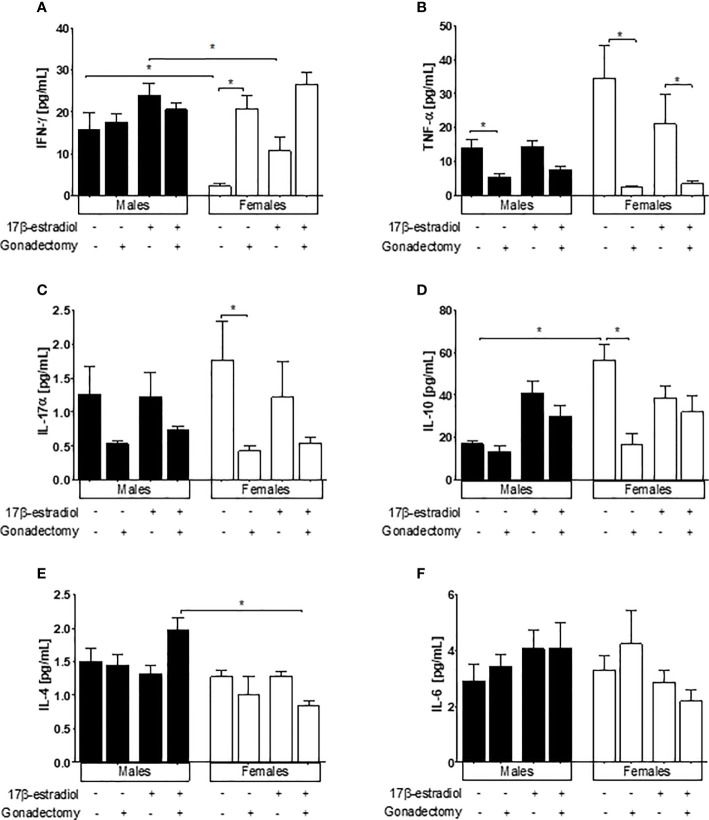
The effect of 17β-estradiol on the concentrations of IFN-γ, TNF-α, IL-4, and IL-10 in the plasma of mice infected with *Plasmodium berghei* ANKA. The concentrations of IFN-γ **(A)**, TNF-α **(B)**, IL-4 **(C)**, IL-10 **(D)**, IL-4 **(E)** and IL-6 **(F)** in the plasma of intact or gonadectomized male and female mice treated with 17β-estradiol and infected with *P. berghei* ANKA were evaluated by flow cytometry. Each bar represents the mean ± SEM. Data are representative of two independent experiments, each using five mice (*n* = 10). Asterisks indicate significant differences between selected groups (*p≤* 0.05) calculated using Kruskal-Wallis test with a post-hoc Dunn’s test.

Intact male mice displayed significantly higher levels of IFN-γ than intact females. Gonadectomy significantly increased IFN-γ concentrations in female mice but did not affect IFN-γ concentrations in their male counterparts. Intact male mice treated with 17β-estradiol displayed higher IFN-γ concentrations than intact female mice in the same condition (**Figure 6A**). These data indicated that 17β-estradiol had a sexually dimorphic effect on IFN-γ concentrations.

The TNF-α concentration was in trend higher in intact female mice than in intact males, gonadectomy significantly decreased TNF-α levels in in both sexes. 17β-estradiol treatment did not modify the levels of TNF-α in gonadectomized or intact mice of either sex (**Figure 6B**).

Gonadectomy significantly decreased the concentration of IL-17α only in female mice; however, 17β-estradiol administration did not alter the levels of IL-17α in gonadectomized mice of either sex (**Figure 6C**). On the other hand, a dimorphic pattern was observed for IL-10, whereby intact male mice exhibited lower concentrations of this cytokine than intact females, while gonadectomy significantly decreased IL-10 concentrations only in female mice. 17β-estradiol treatment did not modify IL-10 levels, either in intact or in gonadectomized mice (**Figure 6D**).

The concentration of IL-4 was not affected by the administration of 17β-estradiol to either sex; however 17β-estradiol administration elicited a sexually dimorphic pattern for IL-4, in which gonadectomized male mice showed higher levels of this cytokine than gonadectomized females (**Figure 6E**). Finally, IL-6 levels did not respond to 17β-estradiol treatment either in intact or in gonadectomized mice (**Figure 6F** **)**.

### In *P. berghei* ANKA-Infected Mice, Gonadectomy Upregulated Cytokine-Related mRNA Expression Levels Only in the Brains of Males

As the infection of CBA/Ca mice with *P. berghei* ANKA is the gold standard model for the investigation of cerebral malaria (21), we also analyzed mRNA expression levels of *Tnf*, *Il1b*, and *Il10* in the brains of male and female mice treated with 17β-estradiol.

**Figure 7:**
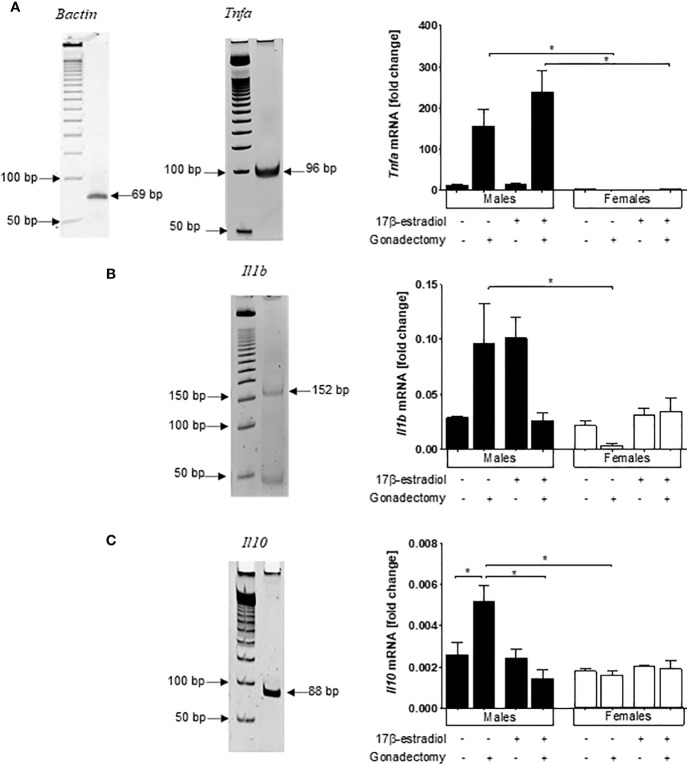
Dimorphic effects of 17β-estradiol on mRNA expression levels of *Tnf*, *Il1b*, and *Il10* in the brains of mice infected with *Plasmodium berghei* ANKA. Intact or gonadectomized male or female mice were treated with 17β-estradiol and infected with *P. berghei* ANKA. On day 8 post-infection, RNA was isolated from brain samples, reverse-transcribed, and subjected to qPCR to quantify the mRNA expression relative to β-actin of *Tnf* **(A)**, *Il1b* **(B)**, and *Il10* **(C)**. Representative acrylamide gel image showing the 50 bp molecular marker and the corresponding gene amplicon size; histograms represent the mean values ± SEM. Data are representative of two independent experiments, each using five mice (*n* = 10) of the relative mRNA expression for the cytokine related to that of β-actin. Asterisks indicate significant differences between selected groups (*P≤* 0.05) calculated using Kruskal-Wallis test with a post -hoc Dunn’s test.

Females exhibited lower *Tnf* mRNA expression than males. Gonadectomized males exhibited higher *Tnf* mRNA expression than gonadectomized females generating a dimorphic pattern; however, neither gonadectomy nor 17β-estradiol administration altered the *Tnf* mRNA expression levels in females (**Figure 7A** **).**

Gonadectomy and 17β-estradiol treatment both led to a no significant increase in the mRNA expression levels of *Il1b* only in male mice generating a dimorphic pattern. Moreover, 17β-estradiol administration to gonadectomized mice significantly decreased the mRNA expression of this cytokine, but again only in males (**Figure 7B**). Finally, for *Il10*, gonadectomy increased the mRNA expression of this gene only in male mice generating once again a dimorphic pattern. However, 17β-estradiol treatment to gonadectomized male mice restored the original *Il10* mRNA expression levels (**Figure 7C**).

### In Mice Infected With *P. berghei* ANKA, Only the Males Showed Reduced Antibody Levels Following Gonadectomy

**Figure 8:**
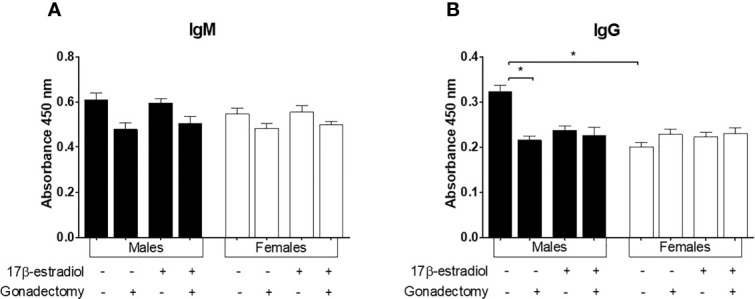
The effect of 17β-estradiol on IgM and IgG levels in mice infected with *Plasmodium berghei* ANKA. Intact or gonadectomized male or female mice were treated with 17β-estradiol and infected with *P. berghei* ANKA. On day 8 post-infection, plasma was obtained and used to quantify IgM **(A)** and IgG **(B)** levels by ELISA. The histograms represent mean values ± SEM. Data are representative of two independent experiments, each using five mice (*n* = 10). Asterisks indicate significative differences between groups (*P* ≤ 0.05) were calculated using Kruskal-Wallis test with a post-hoc Dunn’s test.

17β-estradiol is known to promote antibody production in mice (22). Therefore, we assessed the effect of 17β-estradiol on IgM and IgG antibody levels. Neither gonadectomy nor 17β-estradiol administration modified the IgM concentration. (**Figure 8A**). Interestingly, intact males developed higher levels of IgG antibodies than intact females generating a dimorphic pattern. Gonadectomy significantly decreased the levels of IgG only in males, whereas 17β-estradiol administration did not alter the concentrations of IgG in intact mice (**Figure 8B**).

## Discussion

The results of this study showed that 17β-estradiol triggered a sexually dimorphic pattern in parasitemia, body mass change, temperature, and hemoglobin concentrations. Additionally, a dimorphic pattern was also generated for both CD8^+^ T and NK1.1^+^ cells in the spleen. Finally, 17β-estradiol differentially modified plasma cytokine levels and the mRNA expression of *Tnf*, and *Il1b* in the brain of mice infected with *P. berghei* ANKA.

The administration of 17β-estradiol increased parasitemia in intact female mice. These results corroborate those of Benten et al. (23) for female mice infected with *P. chabaudi*, but are in contrast to those of Klein et al., where no changes in parasitemia were observed in C57Bl/6 female mice treated with 17β-estradiol (14). This contradictory result may be explained by the different doses used. The dose used by Klein et al. was five-fold lower than that used by Benten et al. and by us in this study, and the effects of 17β-estradiol are known to be dose-dependent; low levels of 17β-estradiol promote a Th1 response, whereas high levels enhance IL-10 production (24). A possible explanation for the higher parasitemia recorded in intact female mice treated with 17β-estradiol is that this group exhibited decreased plasma IFN-γ levels compared with their male counterparts. This result corresponds to the increased anti-inflammatory IL-10 levels detected in this group; these findings are critical because IFN-γ is necessary to eliminate the malaria parasite (25). Moreover, the higher parasitemia may probably have resulted from the 17β-estradiol-mediated down-regulation of the levels of pro-inflammatory cytokines through the inhibition of NF-κB-associated transcription factor (26); this particular mechanism, however, should be experimentally demonstrated in our model. Additionally, another possibility is that 17β-estradiol could induce the expansion of regulatory T cells and increase their suppressive effects in the immune response, as described by Polanczyk et al. (27).

The administration of 17β-estradiol to intact males prevented the body mass loss, while it induced mass loss in intact females. Interestingly, the decrease of this steroid due to gonadectomy increased body mass in females but decreased it in males. These findings suggest that the effect of 17β-estradiol on body mass depends on both estradiol concentration and sex. It has been shown that 17β-estradiol upregulates TNF-α synthesis (28), and this cytokine inhibits adipocyte differentiation and promotes lipolysis (29). In this study, gonadectomy reduced TNF-α concentration, which would explain the increment in body mass in gonadectomized female mice. Additionally, the administration of 17β-estradiol to gonadectomized female mice decrease their body mass. Accordingly, Bhardwaj et al. showed that 17β-estradiol administration to gonadectomized female mice induced weight loss (30).

In contrast to human malaria, murine malaria induces hypothermia. In this study, intact females regulated their temperature better than males. Unexpectedly, the administration of 17β-estradiol to intact mice increased temperature in males, but in females, it decreased. Additionally, gonadectomy decreased body temperature in males but increased it in females. These findings suggest that 17β-estradiol helps to regulate the temperature in both sexes.

The decreased Hb concentration (anemia) is another malaria hallmark. The administration of 17β-estradiol decreased the Hb concentration in males but increased it in females. A probable explanation for this finding is that hematopoietic stem cells express ERα (whose numbers are different between sexes) and the interaction 17β-estradiol and ERα signaling promotes erythropoiesis, as shown by Nakada, et al. (31).

In this study, we detected sexual dimorphism in the levels of CD8^+^ T cells in intact mice, with females displaying higher percentages of CD8^+^ cells than intact males. Podoba et al. demonstrated that CD8^+^ T cells are essential during malaria infection. Their depletion delays the clearance of *P. chabaudi* in mice (32). Moreover, CD8^+^ T cells upregulate *Ifng* and *Tnf* mRNA expression in *P. chabaudi* infection (33), with both cytokines playing a role in the macrophage-mediated destruction of the *Plasmodium* parasite. Our results suggested that CD8^+^ T cells contribute to sexual dimorphism in malaria-infected mice; however, 17β-estradiol is likely not to be the only molecule responsible for this sexual dimorphism, because its administration to intact or gonadectomized mice did not affect this cell population. Further studies are required to determine the mechanism underlying the role of 17β-estradiol in this dimorphic pattern.

We also detected a sexual dimorphism for the levels of NK1.1^+^ cells, with intact males exhibiting higher percentages of these cells than females. Following gonadectomy, there was a decrease in the number of NK1.1^+^ cells, while 17β-estradiol administration restored the percentages of these cells to their original levels in both sexes. These findings suggest that NK1.1^+^ cells are regulated by 17β-estradiol in our rodent model of malaria. This finding is important because NK1.1^+^ cells exhibit cytotoxic activity and produce IFN-γ in response to *Plasmodium* infection, which is critical to increasing the phagocytosis of malaria parasites (34).

The plasma levels of IFN-γ, TNF-α, and IL-10 displayed a sex-dependent pattern. The concentrations of these cytokines were modulated after gonadectomy only in female mice, likely due to the consequent reduction in 17β-estradiol levels. Additionally, gonadectomized female mice displayed increased concentrations of IFN-γ and reduced levels of both TNF-α and IL-10. Our results agree with those of Klein et al., who found that 17β-estradiol can modify both IFN-γ and IL-10 levels in *P. chabaudi*-infected female mice (4, 14). Nevertheless, in our study, the administration of 17β-estradiol to gonadectomized female mice did not restore the original levels of these cytokines. These findings suggest that, although 17β-estradiol play a role, there must be other factors produced in the gonads that regulate the plasma levels of these cytokines.

Because cytokines TNF-α and IL-1β contribute to cerebral malaria pathogenesis in mice (35, 36), we also evaluated the effect of 17β-estradiol on the mRNA expression of pro-inflammatory cytokines in the brain. The mRNA expression of *Tnf*, *Il1b*, and *Il10* exhibited sexual dimorphism. It was interesting to notice the increased levels of *Tnf* and *Il1b* in males which explains, at least in part, the higher mortality in this sex, since both cytokines are associated with cerebral malaria (36). Nevertheless, 17β-estradiol treatment did not restore the original level of these cytokines in gonadectomized mice. These findings suggest that 17β-estradiol is not the only mediator in this pathology.

To our knowledge, this study is the first to comprehensively analyze the effects of 17β-estradiol on different aspects of the immune response in the blood, spleen, and brain of female and male mice infected with *Plasmodium berghei* ANKA. Our results explain, at least in part, the sexual dimorphism associated with the immune response to malaria. Further research is required to understand the mechanisms involved in the sexual dimorphism triggered by sex hormones in the immune response to malaria.

We are aware that when we modified the levels of 17β-estradiol, the levels of the precursor hormones may also have been modified. Consequently, our results may reflect the effects of steroids other than 17β-estradiol, such as testosterone, progesterone, and DHEA.

In conclusion, we showed that the immune system of male and female mice infected with *Plasmodium berghei* ANKA responds differentially to 17β-estradiol by modifying the concentrations of immune cells, cytokines, and antibodies. Understanding how 17β-estradiol and other steroids induce sex-dependent differences in immune responses to pathogens is critical for developing individualized treatments or vaccines that are more efficient and less toxic for each sex.

## Data Availability Statement 

The raw data supporting the conclusions of this article will be made available by the authors, without undue reservation.

## Ethics Statement

The animal study was reviewed and approved by Institutional Care and Animal Use Committee of the University (certificate number 28/04/SO/3.4.1) and rigorously adhered to the Mexican Official Guidelines (NOM-062-ZOO-1999) for the use and care of laboratory animals.

## Author Contributions

JA-C and LC-C: design of experiments, data obtention and discussion of results. FB-G: design of experiments, data processing, analysis and discussion of results. TN-P and ML-P: quantification and analysis of cytokines. OF-R: quantification and analysis of cellular populations. DC-R: analysis and discussion of results. ML-H: conceived the project, designed experiments, performed analysis and discussion of results and wrote the paper. All authors contributed to the article and approved the submitted version.

## Funding

This work was supported by the PAPIIT UNAM grants (IN220417 and IN228620) awarded to ML-H. LC-C, JA-C, FB-G and TN-P are CONACyT fellows from the Posgrado en Ciencias Biológicas, UNAM. ML-P is a CONACyT fellow from the Posgrado en Inmunología, ENCB, IPN.

## Conflict of Interest

The authors declare that the research was conducted in the absence of any commercial of financial relationships that could be construed as a potential conflict of interest.

## References

[1] Conroy AL Datta D John CC. What causes severe malaria and its complications in children? Lessons learned over the past 15 years. BMC Med (2019) 17(1):52. 10.1186/s12916-019-1291-z 30841892 PMC6404293

[2] Landgraf B Kollaritsch H Wiedermann G Wernsdorfer WH. Parasite density of Plasmodium falciparum malaria in Ghanaian schoolchildren: evidence for influence of sex hormones? Trans R Soc Trop Med Hyg (1994) 88(1):73–4. 10.1016/0035-9203(94)90505-3 8154009

[3] Zhang Z Chen L Saito S Kanagawa O Sendo F. Possible modulation by male sex hormone of Th1/Th2 function in protection against Plasmodium chabaudi chabaudi AS infection in mice. Exp Parasitol (2000) 96(3):121–9. 10.1006/expr.2000.4572 11162362

[4] Cernetich A Garver LS Jedlicka AE Klein PW Kumar N Scott AL. Involvement of gonadal steroids and gamma interferon in sex differences in response to blood-stage malaria infection. Infect Immun (2006) 74(6):3190–203. 10.1128/IAI.00008-06 PMC1479253 16714546

[5] Kovats S. Estrogen receptors regulate innate immune cells and signaling pathways. Cell Immunol (2015) 294(2):63–9. 10.1016/j.cellimm.2015.01.018 PMC4380804 25682174

[6] Beato M Chavez S Truss M. Transcriptional regulation by steroid hormones. Steroids (1996) 61(4):240–51. 10.1016/0039-128x(96)00030-x 8733009

[7] Ueda K Karas RH. Emerging evidence of the importance of rapid, non-nuclear estrogen receptor signaling in the cardiovascular system. Steroids (2013) 78(6):589–96. 10.1016/j.steroids.2012.12.006 23276634

[8] Grimaldi CM Cleary J Dagtas AS Moussai D Diamond B. Estrogen alters thresholds for B cell apoptosis and activation. J Clin Invest (2002) 109(12):1625–33. 10.1172/JCI14873 PMC151010 12070310

[9] Curran EM Berghaus LJ Vernetti NJ Saporita AJ Lubahn DB Estes DM. Natural killer cells express estrogen receptor-alpha and estrogen receptor-beta and can respond to estrogen via a non-estrogen receptor-alpha-mediated pathway. Cell Immunol (2001) 214(1):12–20. 10.1006/cimm.2002.1886 11902825

[10] Lelu K Laffont S Delpy L Paulet PE Perinat T Tschanz SA. Estrogen receptor alpha signaling in T lymphocytes is required for estradiol-mediated inhibition of Th1 and Th17 cell differentiation and protection against experimental autoimmune encephalomyelitis. J Immunol (2011) 187(5):2386–93. 10.4049/jimmunol.1101578 21810607

[11] Calippe B Douin-Echinard V Laffargue M Laurell H Rana-Poussine V Pipy B. Chronic estradiol administration in vivo promotes the proinflammatory response of macrophages to TLR4 activation: involvement of the phosphatidylinositol 3-kinase pathway. J Immunol (2008) 180(12):7980–8. 10.4049/jimmunol.180.12.7980 18523261

[12] Benten WP Wunderlich F Mossmann H. Plasmodium chabaudi: estradiol suppresses acquiring, but not once-acquired immunity. Exp Parasitol (1992) 75(2):240–7. 10.1016/0014-4894(92)90184-C 1516672

[13] Libonati RM Cunha MG Souza JM Santos MV Oliveira SG Daniel-Ribeiro CT. Estradiol, but not dehydroepiandrosterone, decreases parasitemia and increases the incidence of cerebral malaria and the mortality in plasmodium berghei ANKA-infected CBA mice. Neuroimmunomodulation (2006) 13(1):28–35. 10.1159/000093271 16699290

[14] Klein PW Easterbrook JD Lalime EN Klein SL. Estrogen and progesterone affect responses to malaria infection in female C57BL/6 mice. Gend Med (2008) 5(4):423–33. 10.1016/j.genm.2008.10.001 PMC4155322 19108815

[15] Grau GE Piguet PF Vassalli P Lambert PH. Tumor-necrosis factor and other cytokines in cerebral malaria: experimental and clinical data. Immunol Rev (1989) 112:49–70. 10.1111/j.1600-065x.1989.tb00552.x 2575074

[16] Legorreta-Herrera M Mosqueda-Romo NA Nava-Castro KE Morales-Rodriguez AL Buendia-Gonzalez FO Morales-Montor J. Sex hormones modulate the immune response to Plasmodium berghei ANKA in CBA/Ca mice. Parasitol Res (2015) 114(7):2659–69. 10.1007/s00436-015-4471-6 25876048

[17] Aguilar-Castro J Cervantes-Candelas LA Buendia-Gonzalez FO Nolasco-Perez TJ Lopez-Padilla MS Fernández-Rivera O. Dimorphic effect of 17beta-estradiol on pathology and oxidative stress in experimental malaria. Immunobiology (2020) 225(1):151873. 10.1016/j.imbio.2019.11.008 31812344

[18] Legorreta-Herrera M Ventura-Ayala ML Licona-Chavez RN. Soto-Cruz IHernandez-Clemente FF. Early treatment during a primary malaria infection modifies the development of cross immunity. Parasite Immunol (2004) 26(1):7–17. 10.1111/j.0141-9838.2004.00677.x 15198641

[19] Svechnikov K Soder O. Ontogeny of gonadal sex steroids. Best Pract Res Clin Endocrinol Metab (2008) 22(1):95–106. 10.1016/j.beem.2007.09.002 18279782

[20] Henry B Roussel C Carucci M Brousse V Ndour PA Buffet P. The Human Spleen in Malaria: Filter or Shelter? Trends Parasitol (2020) 36(5):435–46. 10.1016/j.pt.2020.03.001 32298631

[21] de Souza JB Riley EM. Cerebral malaria: the contribution of studies in animal models to our understanding of immunopathogenesis. Microbes Infect (2002) 4(3):291–300. 10.1016/s1286-4579(02)01541-1 11909739

[22] Aguilar-Pimentel JA Cho YL Gerlini R Calzada-Wack J Wimmer M Mayer-Kuckuk D. Increased estrogen to androgen ratio enhances immunoglobulin levels and impairs B cell function in male mice. Sci Rep (2020) 10(1):18334. 10.1038/s41598-020-75059-9 33110090 PMC7591566

[23] Benten WP Wunderlich F Herrmann R Kuhn-Velten WN. Testosterone-induced compared with oestradiol-induced immunosuppression against Plasmodium chabaudi malaria. J Endocrinol (1993) 139(3):487–94. 10.1677/joe.0.1390487 8133215

[24] De Leon-Nava MA Nava K Soldevila G Lopez-Griego L Chavez-Rios JR Vargas-Villavicencio JA. Immune sexual dimorphism: effect of gonadal steroids on the expression of cytokines, sex steroid receptors, and lymphocyte proliferation. J Steroid Biochem Mol Biol (2009) 113(1-2):57–64. 10.1016/j.jsbmb.2008.11.003 19073259

[25] Shear HL Srinivasan R Nolan T Ng C. Role of IFN-gamma in lethal and nonlethal malaria in susceptible and resistant murine hosts. J Immunol (1989) 143(6):2038–44. 2506274

[26] McCracken SA Gallery E Morris JM. Pregnancy-specific down-regulation of NF-kappa B expression in T cells in humans is essential for the maintenance of the cytokine profile required for pregnancy success. J Immunol (2004) 172(7):4583–91. 10.4049/jimmunol.172.7.4583 15034076

[27] Polanczyk MJ Hopke C Huan J Vandenbark AA Offner H. Enhanced FoxP3 expression and Treg cell function in pregnant and estrogen-treated mice. J Neuroimmunol (2005) 170(1-2):85–92. 10.1016/j.jneuroim.2005.08.023 16253347

[28] Zaldivar V Magri ML Zarate S Jaita G Eijo G Radi D. Estradiol increases the expression of TNF-alpha and TNF receptor 1 in lactotropes. Neuroendocrinology (2011) 93(2):106–13. 10.1159/000323760 21252492

[29] Wang H Ye J. Regulation of energy balance by inflammation: common theme in physiology and pathology. Rev Endocr Metab Disord (2015) 16(1):47–54. 10.1007/s11154-014-9306-8 25526866 PMC4346537

[30] Bhardwaj P Du B Zhou XK Sue E Giri D Harbus MD. Estrogen Protects against Obesity-Induced Mammary Gland Inflammation in Mice. Cancer Prev Res (Phila) (2015) 8(8):751–9. 10.1158/1940-6207.CAPR-15-0082 PMC4526346 26038116

[31] Nakada D Oguro H Levi BP Ryan N Kitano A Sitoh Y. Oestrogen increases haematopoietic stem-cell self-renewal in females and during pregnancy. Nature (2014) 505(7484):555–8. 10.1038/nature12932 PMC4015622 24451543

[32] Podoba JE Stevenson MM. CD4+ and CD8+ T lymphocytes both contribute to acquired immunity to blood-stage Plasmodium chabaudi AS. Infect Immun (1991) 59(1):51–8. 10.1128/IAI.59.1.51-58.1991 PMC257704 1898902

[33] Legorreta-Herrera M Rivas-Contreras S Ventura-Gallegos J Zentella-Dehesa A. Nitric oxide is involved in the upregulation of IFN-gamma and IL-10 mRNA expression by CD8(+) T cells during the blood stages of P. chabaudi AS infection in CBA/Ca mice. Int J Biol Sci (2011) 7(9):1401–11. 10.7150/ijbs.7.1401 PMC3221947 22110391

[34] Korbel DS Finney OC Riley EM. Natural killer cells and innate immunity to protozoan pathogens. Int J Parasitol (2004) 34(13-14):1517–28. 10.1016/j.ijpara.2004.10.006 15582528

[35] Hunt NH Ball HJ Hansen AM Khaw LT Guo J Bakmiwewa S. Cerebral malaria: gamma-interferon redux. Front Cell Infect Microbiol (2014) 4:113. 10.3389/fcimb.2014.00113 25177551 PMC4133756

[36] Armah H Wired EK Dodoo AK Adjei AA Tettey Y Gyasi R. Cytokines and adhesion molecules expression in the brain in human cerebral malaria. Int J Environ Res Public Health (2005) 2(1):123–31. 10.3390/ijerph2005010123 PMC3814706 16705810

